# Do We Belittle Essential Tremor by Calling It a Syndrome Rather Than a Disease? No

**DOI:** 10.3389/fneur.2020.586606

**Published:** 2020-09-30

**Authors:** Rodger J. Elble

**Affiliations:** Department of Neurology, Southern Illinois University School of Medicine, Springfield, IL, United States

**Keywords:** essential tremor, classification, diagnostic axes, tremor, syndrome

## Abstract

A task force of the International Parkinson and Movement Disorder Society (MDS) recently published a tremor classification scheme that is based on the nosologic principle of two primary axes for classifying an illness: clinical manifestations (Axis 1) and etiology (Axis 2). An Axis 1 clinical syndrome is a recurring group of clinical symptoms, signs (physical findings), and possibly laboratory results that suggests the presence of at least one underlying Axis 2 etiology. Syndromes must be defined and used consistently to be of value in finding specific etiologies and effective treatments. The MDS task force concluded that essential tremor is a common neurological syndrome that has never been defined consistently by clinicians and researchers. The MDS task force defined essential tremor as a syndrome of bilateral upper limb action tremor of at least 3 years duration, with or without tremor in other locations (e.g., head, voice, or lower limbs), in the absence of other neurological signs (e.g., dystonia, parkinsonism, myoclonus, ataxia, peripheral neuropathy, and cognitive impairment). Deviations from this definition should not be labeled as essential tremor. Patients with additional questionably-abnormal signs or with signs of uncertain relevance to tremor are classified as essential tremor plus. The MDS classification scheme encourages a thorough unbiased phenotyping of patients with tremor, with no assumptions of etiology, pathology, pathophysiology, or relationship to other neurological disorders. The etiologies, pathology, and clinical course of essential tremor are too heterogeneous for this syndrome to be viewed as a disease or a family of diseases.

## Introduction

An international task force on tremor was convened by the International Parkinson and Movement Disorder Society (MDS) in 2011 to review the 1998 MDS consensus statement on tremor, which was devoted to the classification of pathologic tremors (1). The Task Force found that the 1998 consensus did not use a consistent approach to tremor classification. Tremor classifications were variably based on presumed anatomical origin (e.g., cerebellar tremor), presumed etiology (e.g., neuropathic tremor), and clinical phenomenology (e.g., primary writing tremor, isolated voice tremor). The Task Force was concerned that essential tremor (ET) was often viewed as a specific disease, rather than a clinical syndrome, and that ET was not defined and diagnosed consistently in the clinic or in research. A revised classification scheme (2) emerged from a comprehensive review of the literature and 5 years of intense discussion that included four 1-h meetings, a 2-day conference, several teleconferences, and numerous e-mail exchanges and document drafts. The revised classification scheme is based on the nosologic principle of two primary axes for classifying an illness: clinical manifestations (Axis 1) and etiology (Axis 2) (3). The clinical manifestations in Axis 1 include symptoms, signs, and laboratory results that characterize the tremor disorder.

## Essential Tremor Is a Syndrome

A syndrome is a recurring group of Axis 1 clinical symptoms, signs (physical findings), and possibly laboratory results that suggests the presence of at least one underlying etiology (4). The Task Force acknowledged the existence of many useful Axis 1 tremor syndromes and broadly defined two groups of tremor syndromes: those in which tremor is the only abnormal sign (isolated tremor syndromes) and those in which tremor occurs in combination with one or more additional signs such as dystonia or ataxia (combined tremor syndromes). ET was originally viewed as “a tremor diathesis that was often familial and occurred in isolation of other neurologic signs” (5). The Task Force concluded that this view of ET is still valid and formally defined ET as an isolated tremor syndrome of bilateral upper limb action tremor of at least 3 years duration, with or without tremor in other locations (e.g., head, voice, or lower limbs). This definition of ET differs from the widely-used TRIG criteria (Tremor Investigation Group) only in the required 3-year history of tremor, instead of 5 years (1). The MDS definition of ET characterizes the vast majority of people with ET, most of whom have not seen a physician for their tremor (6, 7). These people have a long-standing, relatively-mild ET syndrome (8, 9) with strong heritability (10).

## The Value of Clinical Syndromes

Syndromes are useful only to the extent that they facilitate the discovery of useful treatments and specific etiologies, and by this standard, the syndrome of ET has been disappointing. The Task Force debated extensively whether ET should be defined more broadly or more narrowly, but ultimately, no conclusion was possible because the syndrome of ET has never been defined and used consistently (11). ET has been used loosely to include tremor syndromes ranging from enhanced physiologic tremor to action tremor in patients with neurological diseases such as Parkinson disease (12). Louis (13) has referred to ET as a “family of diseases” with an “evolving definition” (13) and “premotor stage” (14). The validity of these concepts is questionable. The pathologic and etiologic heterogeneities of ET are so great that the concept of “family” has no validity. An “evolving definition” of ET is precisely what the MDS Task Force wanted to avoid. The Task Force encouraged the definition of additional tremor syndromes within Axis 1 if these syndromes are believed to be useful in defining cohorts of patients that lead to the identification of specific Axis 2 etiologies. However, a clinical syndrome must be defined and used consistently to be of value in the discovery of useful treatments and specific etiologies. Inconsistent “evolving” definitions of ET make published studies difficult or impossible to reconcile. Misdiagnosis is understandably common (15–17), even among movement disorder specialists (18).

ET is defined as an isolated tremor syndrome in which tremor is the only permissible neurologic sign. A major problem has been that specialists differ in their thresholds for identifying dystonia, Parkinsonism, ataxia, and other neurological signs. Mild neurological abnormalities are commonly missed or dismissed in the evaluation of patients with possible ET. Questionable signs of dystonia such as a mild head tilt, spooning posture of the extended hands (19), and index finger extension (20) occur too commonly in normal people to be used confidently in clinical diagnosis. Jerkiness and asymmetry are features of dystonic tremor (21), but these characteristics have never been operationally defined. Impaired tandem gait in ET patients is often interpreted as a cerebellar sign, but this common test has never been properly validated, making interpretation difficult, particularly in the elderly (22). The Task Force concluded that questionably abnormal clinical manifestations should be consistently documented and that ET plus should be the classification of patients who fulfill the criteria for ET but have one or more of these “soft” signs of uncertain significance (2). The classification ET plus encourages clinicians to document all deviations from the ET syndrome that are of questionable significance (e.g., spooning of the hands, unsteady tandem gait) or questionable relevance to tremor (e.g., mild cognitive impairment).

Retrospective reviews of outpatient clinical cohorts have shown that 40% or more of patients previously diagnosed as ET are reclassified as ET plus or a combined tremor syndrome when the new MDS classification scheme is applied (23–25). For example, 20 of the last 34 patients undergoing DBS surgery for ET at our center were reclassified as ET plus due to the following Axis 1 features: rest tremor or questionable rest tremor (*n* = 9), questionable dystonic posturing (*n* = 14), jerky tremor (*n* = 7), asymmetry in upper limb tremor exceeding 1 point on the Essential Tremor Rating Assessment Scale (*n* = 8) (26), rapid progression (*n* = 6), strained voice (*n* = 3), and impaired tandem gait (*n* = 7). These changes in diagnosis cannot be attributed to a drastic change in the definition of ET because the new definition of ET differs from the old TRIG definition only in the required duration of tremor (3 vs. 5 years) and differs from the old MDS consensus criteria only in the exclusion of isolated head tremor and the required 3-year history of tremor. Instead, the changes in diagnosis are primarily due to the new classification ET plus, which places great emphasis on documenting additional signs of uncertain abnormality and relevance to tremor. Previously, these additional signs were frequently overlooked, not documented, or wrapped into the diagnosis ET.

There is already evidence that the deeper phenotyping inherent in ET plus is worthwhile. Merchant et al. (27) found that patients with signs of ataxia were more likely to develop rapid tolerance to thalamic deep brain stimulation, and Picillo et al. (28) found that patients with ET plus were more likely to develop dystonia from thalamic neurosurgery. Geneticists are also beginning to embrace this approach to tremor classification (29).

## The Limitations of Clinical Syndromes

The classifications ET and ET plus are purely clinical, and it is recognized that experts will disagree on the Axis 1 classification of patients, particularly those patients who are older and have greater tremor severity (18, 30). The presence of one questionably-abnormal sign, such as three missteps in a 10-step tandem walk, may not be deemed sufficient to exclude an older person from a therapeutic trial of ET but will likely reach the threshold for ET plus in a 20-year old with no other medical problems. A patient that is completely unable to tandem walk and is also unsteady when walking should be classified as having a combined tremor-ataxia syndrome, not ET or ET plus. Similarly, spooning hand posturing alone could be a normal variant, but spooning in combination with jerky asymmetric upper limb tremor [≥1 point on the Essential Tremor Rating Assessment Scale (26)] may be regarded as too suggestive of dystonic tremor to be classified as ET or ET plus. True rest tremor occurs in <15% of clinic patients (31) and in <5% of people in the general population who otherwise fulfill criteria for ET (32). Therefore, the MDS Task Force concluded that patients meeting the criteria for ET except for the presence of rest tremor should be classified as ET plus. These uncertainties illustrate that many aspects of the neurological exam are still in need of validation and standardization. Clinical constructs such as jerkiness, unsteadiness, and asymmetry need to be operationally defined and quantified, possibly with the aid of quantitative motion analysis and clinical electrophysiology (21).

**Figure 1:**
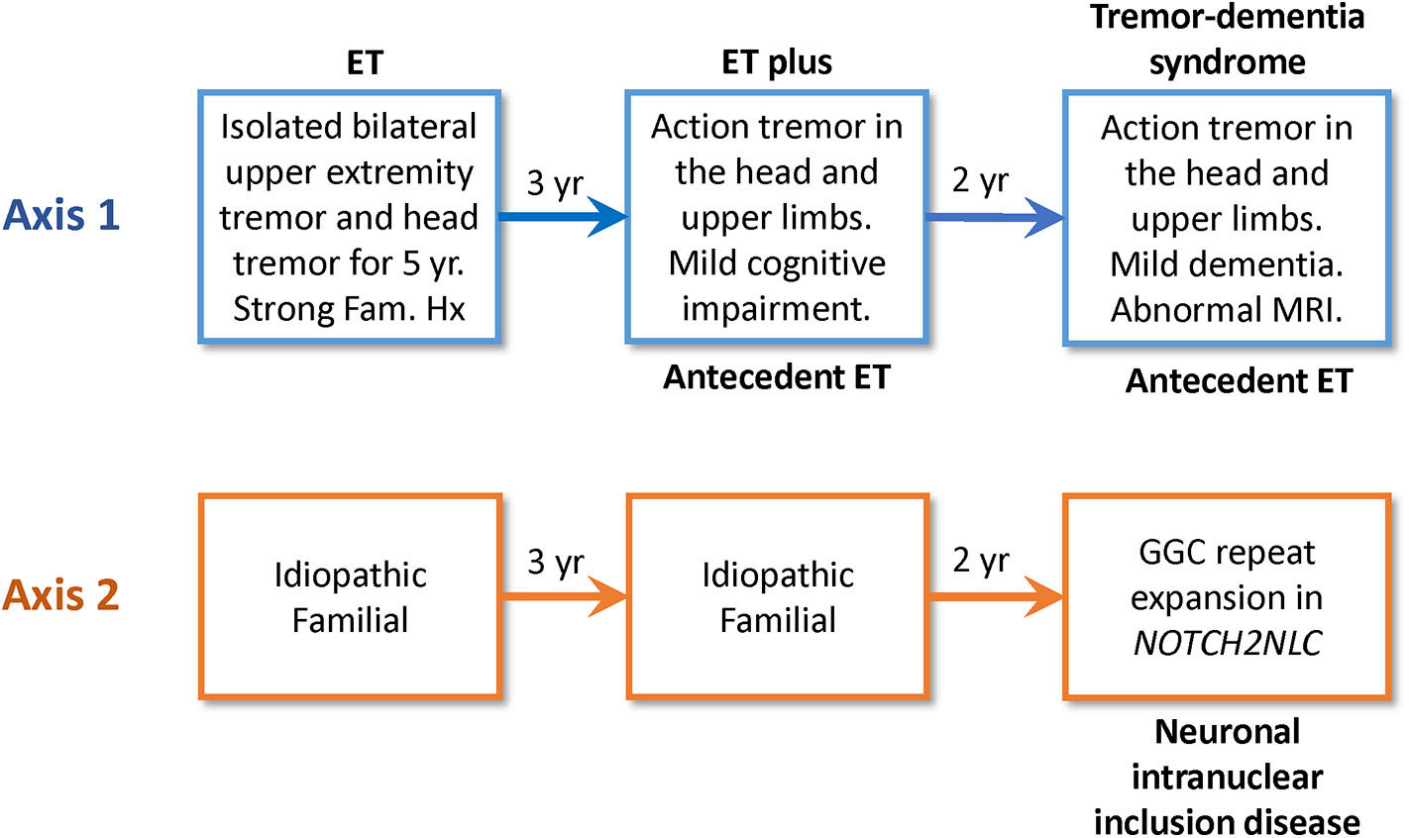
This flow diagram illustrates how Axis 1 and 2 classifications may change over time. This clinical scenario is based on the work of Chen et al. (40). A 54-year-old Chinese man presented with a 5-year history of tremor in the head and upper limbs. His family history was consistent with autosomal dominant inheritance. His initial Axis 1 classification was ET, and his Axis 2 classification was idiopathic familial. Over time, his Axis 1 classification changed from ET to ET plus mild cognitive impairment, and his Axis 1 classification ultimately changed to a combined tremor-dementia syndrome with antecedent ET. His MRI brain revealed diffusion-weighted abnormality in the subcortical U-fibers of both frontal lobes, and genetic testing revealed a GGC repeat expansion in *NOTCH2NLC*. Thus, his Axis 2 diagnosis was ultimately neuronal intranuclear inclusion disease that presented initially as ET.

One criticism of the new MDS classification scheme is that ET and ET plus are diagnostic placeholders, not final diagnoses or specific diseases (33). However, this is true of all medical conditions that are defined solely in terms of clinical manifestations (Axis 1) and not etiology (Axis 2). Clinical syndromes (e.g., acquired immunodeficiency syndrome, AIDS) are useful only to the extent that they facilitate the discovery of specific etiologies (human immunodeficiency virus, HIV) and effective treatments (antiretroviral drugs). A disease is not discovered until the underlying etiology is identified. Furthermore, a patient's syndrome or condition may change as the disease progresses. Thus, ET and ET plus may evolve into a more complex (combined) tremor syndrome before an Axis 2 etiology is discovered. Such patients are then classified with their Axis 2 etiology and current Axis 1 tremor syndrome and are said to have antecedent ET or ET plus (Figure 1).

ET can be a stable syndrome throughout a person's life, given the presence of this syndrome in many patients with a decades-long history of tremor. The stipulated 3-year history of tremor is an attempt, admittedly arbitrary, to increase the likelihood of a stable clinical syndrome. It is widely acknowledged that longitudinal studies are needed to determine the degree to which the ET syndrome is stable (34) and to determine the significance of a stable ET syndrome in terms of underlying etiology and pathophysiology.

## Etiologies of the Essential Tremor Syndrome

ET has an additive heritability of at least 75%, so environmental factors probably play a small and still undefined role (35). Large families with apparent Mendelian dominant inheritance are common, but after more than 25 years of extensive searching, only four genes with rare causative mutations have been discovered: fused in sarcoma gene (*FUS*) (36), GGC repeat expansion in the Notch 2 N-terminal like C gene (*NOTCH2NLC*) (37), HtrA Serine Peptidase 2 gene (*HTRA2*) (38), and teneurin transmembrane protein 4 gene (*TENM4*) (39). There is little doubt that others will be discovered. However, these rare causative mutations are not found in most ET patients. Moreover, studies of families with these mutations illustrate the important fact that ET is frequently not a stable phenotype. ET can be the initial phenotype of neuronal intranuclear inclusion disease (GGC repeat expansion in the *NOTCH2NLC* gene) (40) but may evolve into a more complex syndrome with dementia, parkinsonism, ataxia, convulsions, neuropathy, or autonomic dysfunction (41) (Figure 1). ET may exist for years before a patient with the *HTRA2 p.G399S* allele develops Parkinsonism (38). ET is also an early but temporary phenotype of hereditary dystonia (e.g., *ANO3*) (42), hereditary ataxia (e.g., SCA12) (43), and PARK-*parkin* disease (44). Progression of these diseases ultimately produces complex combined tremor syndromes. In summary, ET is a syndrome or phenotype with many genetic etiologies. Monogenic inheritance appears to be rare, and polygenic or epigenetic inheritance may be a factor, even in families with rare causative gene mutations (39). The genetic heterogeneity of ET seems inconsistent with the notion that ET is “a family of diseases.”

Purkinje cell pathology is found in some but not all ET patients (45–47). However, comparable Purkinje cell loss is also found in diseases that do not cause tremor, such as Huntington disease (48) and Alzheimer disease (49). It is unclear whether distinctive cerebellar pathology is associated with ET (50), and it is also unclear whether the reported Purkinje cell pathology is tremorogenic. The notion that ET is a “Purkinjopathy” belies the etiologic, pathologic, and pathophysiologic complexity of ET (51). Purkinje cell pathology is no justification for regarding ET as “a family of diseases.”

## Pathophysiology of Essential Tremor Syndrome

ET is produced by abnormal oscillation and neuronal entrainment in the cerebellothalamocortical pathway. However, this is true for all forms of pathologic tremor (52). The cerebellum and thalamocortical loop have direct or indirect connections with virtually all motor pathways of the nervous system. Therefore, the source of oscillation in a patient with ET need not be the cerebellum or the thalamocortical loop, and the source may vary among etiologies of ET. Cerebellar Purkinje cells and neurons in the thalamocortical loop have intrinsic membrane properties that are conducive to oscillation (53, 54), and the cerebellum and thalamocortical loop have network properties that could amplify oscillation and promote neuronal entrainment of oscillation originating nearly anywhere in the nervous system (54–56). Oscillation in the cerebellothalamocortical pathway will produce tremor if there is sufficient neuronal entrainment. It is likely that virtually all etiologies of ET produce oscillation in the cerebellothalamocortical pathway. Therefore, the etiologic heterogeneity of ET and syndromic classification of ET should not deter us from conducting therapeutic trials that target the mechanisms of oscillation in the cerebellothalamocortical pathway.

## Subtypes of the Essential Tremor Syndrome

It is possible that the current definition of ET is too broad to identify etiologies and effective treatments. Researchers and clinicians are free to define subtypes of ET, such as late-onset ET (e.g., onset after age 65), familial (e.g., one or more first-degree relatives with ET), sporadic, and tremor predominantly (not exclusively) in the head or voice. However, data from one subtype may not be applicable to all patients with ET. The reasons are obvious. Elderly patients with late-onset action tremor are far more likely to have undiagnosed subclinical neurological comorbidities than young healthy adults (57), and they are more likely to have comorbid systemic illnesses that cause enhanced physiologic tremor, which is easily mistaken for mild ET (6). Familial and sporadic cases are likely to differ in their likelihood of harboring risk genes. Patients with predominant head or voice tremor may be more likely to have a form of dystonia.

It is also possible that the current definition of ET is too narrow to identify etiologies and effective treatments. The MDS classification scheme permits the definition of additional Axis 1 tremor syndromes in which the criteria for ET are met except for the existence of one or more additional Axis 1 features (e.g., gait ataxia). To avoid confusion, these combined tremor syndromes should not be referred to as subtypes or variants of ET.

## Discussion

Syndromes must be defined and used consistently to be of value in clinical care and research. The ET syndrome has never been defined and used consistently. This has made the sizeable literature on ET difficult to interpret because readers must carefully examine each paper for differences in definition that can affect outcome.

The new ET and ET plus classifications do not invalidate earlier studies that carefully defined the axis 1 clinical characteristics of their patient populations, but the results of older studies may need some reinterpretation in the context of the new MDS classification scheme. The main problem with many older studies is that clinicians and researchers commonly used *ad hoc* definitions of ET, and neurological signs of uncertain significance (abnormality) and uncertain relevance to tremor (e.g., mild cognitive impairment in an elderly patient with ET) were often not documented or simply wrapped in a diagnosis of ET. Even patently abnormal signs other than tremor were deemed as permissible within some definitions of ET (58, 59). Furthermore, some studies included isolated head tremor, isolated voice tremor and tremor of <1 year duration (60).

ET plus is a new classification, not a specific syndrome. Clinicians are encouraged to carefully document the additional Axis 1 manifestations beyond tremor when using the classification ET plus. ET plus may include a variety of neurologic signs that are questionably abnormal or questionably relevant to the patient's tremor disorder. Specific ET plus syndromes are permissible within the new classification scheme, as long as the syndromes are defined and used consistently.

A syndrome should not be expanded or changed unless there is good reason to believe that the newly defined syndrome will be a better tool for the discovery of underlying etiology or effective treatment. Changing the definition of a syndrome like ET creates confusion in the comparison of new and old clinical studies. The new MDS definition of the ET syndrome does not differ significantly from the old and widely-used TRIG criteria (1) and is completely compatible with the original concept of ET (5). The new classification ET plus provides us with a tremor classification in which new syndromes can be defined, without disturbing the definition of ET. Subtypes of ET are permissible with the caveat that data from this subtype may not apply to the broad ET patient population.

People with ET and ET plus may be included in the same study cohort if this is believed to facilitate the study objectives. The new MDS definitions of ET and ET plus make no assumptions about underlying etiology or response to treatment. Patients with ET and ET plus may or may not have the same underlying etiology. Furthermore, it is clear that cerebellothalamocortical oscillation is a cornerstone of all forms of tremor, so the notion of ET being a syndrome should not deter one from pursuing new treatments. Careful phenotyping and classification under the new classification scheme will permit *post hoc* exploratory data analyses, and the results can be confirmed or refuted in subsequent studies.

In conclusion, the MDS classification scheme provides much-needed rigor to the classification of ET and puts ET in the proper perspective of being a clinical syndrome, not a specific disease. The classification ET plus facilitates a deeper phenotyping of patients without assumptions of etiology or causality. This should facilitate gene discovery, given the likely polygenic inheritance of ET in most patients. These views do not belittle ET, rather they properly acknowledge the importance of thorough Axis 1 phenotyping, unencumbered by any assumptions of etiology, pathology, or pathophysiology.

## Data Availability Statement

The original contributions presented in the study are included in the article/supplementary material, further inquiries can be directed to the corresponding author/s.

## Author Contributions

The author is solely responsible for the writing and content of this manuscript.

## Conflict of Interest

RE was chair of the Tremor Task Force of the International Parkinson and Movement Disorder Society. He has served as a consultant for Cadent, Cavion, Cydan, Five Microns, Jazz, Merz, Osmotica, Praxis Precision Medicines, and Sage in the design and execution of clinical trials for essential tremor. He serves on advisory boards for the International Essential Tremor Foundation and the Neuroscience Research Foundation of Kiwanis International, Illinois-Eastern Iowa District, and he receives research grant support from this foundation. This article is related to a companion perspective: Do We Belittle Essential Tremor by Calling it a Syndrome Rather than a Disease? Yes. Both Articles were prepared independent of each other.
